# A PilZ-Containing Chemotaxis Receptor Mediates Oxygen and Wheat Root Sensing in *Azospirillum brasilense*

**DOI:** 10.3389/fmicb.2019.00312

**Published:** 2019-03-01

**Authors:** Lindsey O’Neal, Shehroze Akhter, Gladys Alexandre

**Affiliations:** Department of Biochemistry and Cellular and Molecular Biology, The University of Tennessee, Knoxville, Knoxville, TN, United States

**Keywords:** *Azospirillum*, aerotaxis, c-di-GMP, plant-microbe association, PilZ

## Abstract

Chemotactic bacteria sense environmental changes via dedicated receptors that bind to extra- or intracellular cues and relay this signal to ultimately alter direction of movement toward beneficial cues and away from harmful environments. In complex environments, such as the rhizosphere, bacteria must be able to sense and integrate diverse cues. *Azospirillum brasilense* is a microaerophilic motile bacterium that promotes growth of cereals and grains. Root surface colonization is a prerequisite for the beneficial effects on plant growth but how motile *A. brasilense* navigates the rhizosphere is poorly studied. Previously only 2 out of 51 *A. brasilense* chemotaxis receptors have been characterized, AerC and Tlp1, and only Tlp1 was found to be essential for wheat root colonization. Here we describe another chemotaxis receptor, named Aer, that is homologous to the *Escherichia coli* Aer receptor, likely possesses an FAD cofactor and is involved in aerotaxis (taxis in an air gradient). We also found that the *A. brasilense* Aer contributes to sensing chemical gradients originating from wheat roots. In addition to *A. brasilense* Aer having a putative N-terminal FAD-binding PAS domain, it possesses a C-terminal PilZ domain that contains all the conserved residues for binding c-di-GMP. Mutants lacking the PilZ domain of Aer are altered in aerotaxis and are completely null in wheat root colonization and they also fail to sense gradients originating from wheat roots. The PilZ domain of Aer is also vital in integrating Aer signaling with signaling from other chemotaxis receptors to sense gradients from wheat root surfaces and colonizing wheat root surfaces.

## Introduction

The rhizosphere is a chemical milieu, and rhizobacteria must navigate this environment to find habitable niches. Chemotactic bacteria adjust their motility in response to environmental chemicals, swimming toward favorable signals (attractants) and away from harmful chemicals. Chemotaxis receptors drive this recognition either directly, by binding to environmental cues or indirectly, by binding to proteins bound to these cues or by monitoring the effects that exposure to the cues has on metabolism (Wadhams and Armitage, 2004; Schweinitzer and Josenhans, 2010). The latter behavior is typically referred to as energy taxis (Schweinitzer and Josenhans, 2010). Upon recognition of chemoeffectors, chemotaxis receptors transduce this signal to downstream proteins (CheW, CheA, and CheY) that can relay this information to the flagellum machinery and alter the direction of rotation of the flagellum and hence, the swimming direction of the cell. Navigating an attractant gradient prevents changes in the swimming direction, causing the bacteria to keep swimming in the same direction (Adler, 1975). The molecular mechanism of chemotaxis is best known in *Escherichia coli*, and research in diverse Bacteria and Archaea has shown that molecular events during chemotaxis signal transduction are largely conserved across prokaryotes (Briegel et al., 2015). The greatest diversity in chemotaxis signal transduction is seen in the chemotaxis repertoire with bacterial genomes encoding up to 80 chemotaxis receptors (Wuichet and Zhulin, 2010). Many chemotactic bacteria possess multiple chemosensory operons which can control chemotaxis or alternative cellular functions (Wuichet and Zhulin, 2010). Soil bacteria are characterized by both a large number of chemotaxis receptors and multiple chemotaxis signaling systems (Buchan et al., 2010; Scharf et al., 2016), and plant-associated soil bacteria are more likely to encode chemotaxis genes than non-plant associated bacteria (Levy et al., 2018). These features are thought to provide a competitive advantage within the chemically rich and complex environment of the rhizosphere (Scharf et al., 2016).

*Azospirillum brasilense* is a motile, chemotactic plant growth promoting proteobacterium that inhabits the soil and colonizes the roots of diverse plants, including economically important cereals (Pereg et al., 2016). The genome of *A. brasilense* encodes four chemosensory operons, named Che1 through Che4, and 51 chemotaxis receptors (Ulrich and Zhulin, 2010; Mukherjee et al., 2016). Only two of the chemotaxis systems, Che1 and Che4, are directly involved in chemotaxis, and the other two systems have functions outside of chemotaxis, including a likely role in flagellum biosynthesis [Che2 (unpublished data)] and cyst development [Che3 (Bible et al., 2015)]. To date, the function of only two of the chemotaxis receptors have been characterized in this bacterial species: Tlp1 and AerC (Greer-Phillips et al., 2004; Xie et al., 2010; Russell et al., 2013; O’Neal et al., 2017). Both are important in aerotaxis (taxis in an oxygen gradient), but only Tlp1 is necessary for plant root colonization (Greer-Phillips et al., 2004; Xie et al., 2010). Aerotaxis is a major behavioral response in *A. brasilense*, and the bacterium relies on sensing oxygen gradients to locate niches that are best suited for the microaerophilic lifestyle of the bacterium (Barak et al., 1982; Zhulin et al., 1996). AerC is soluble chemotaxis receptor required for aerotaxis under conditions of nitrogen fixation, but not in presence of an organic source of nitrogen (Xie et al., 2010). Tlp1 is a prototypical chemotaxis receptor in that it is a membrane anchored protein with a periplasmic sensory domain at the N-terminus and a cytoplasmic signaling domain. The ligand sensed by Tlp1 is unknown, but the receptor also possesses an additional C-terminal PilZ domain (Russell et al., 2013; O’Neal et al., 2017). PilZ domains are known to bind to the secondary messenger cyclic diguanylate monophosphate (c-di-GMP) (Ryjenkov et al., 2006; Hengge, 2009; Römling et al., 2013). C-di-GMP metabolism is regulated by a variety of cues and has been implicated in modulating the lifestyle of bacteria inhabiting various environments, including the rhizosphere. In *A. brasilense*, c-di-GMP binding to the PilZ domain of Tlp1 regulates receptor sensitivity in an oxygen gradient, and Tlp1 is essential for plant root colonization (Russell et al., 2013; O’Neal et al., 2017). *A. brasilense* encodes several other PilZ containing chemotaxis receptors, each of different predicted topologies. Here, we characterize another PilZ-containing receptor, Aer, and its role in aerotaxis, sensing of root exudate gradients, and root surface colonization.

## Results

### Identification of Chemotaxis Receptors Containing PilZ Domains

**FIGURE 1:**
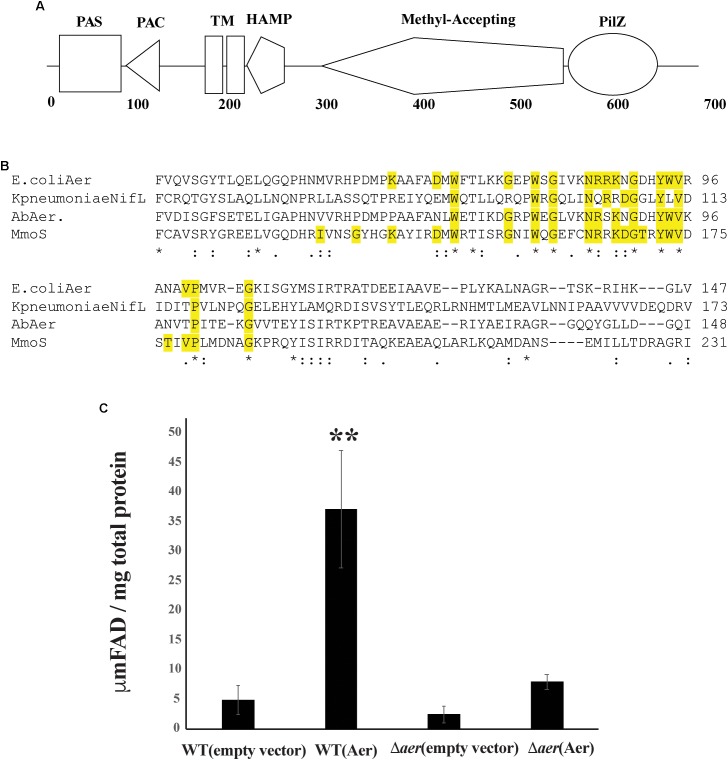
The Aer protein domain organization. **(A)** Protein encoded by AZOBRv2_p280026 contains a PAS, PAC, Transmembrane domains, HAMP, MA, and PilZ domain. **(B)** Conserved residues alignment between AZOBRv2_p280026 (Aer) PAS domains binding FAD in *E. coli* Aer, *Klebsiella pneumoniae* NifL and *Methylococcus capsulatus* Mmos Below the sequence alignment, “^∗^” indicates positions with strictly conserved residues, “:” indicates position with strongly similar residues and “.” indicates positions with weakly similar residues. Yellow highlighted residues are those implicated in FAD binding (Schmitz, 1997; Bibikov et al., 2000; Ukaegbu and Rosenzweig, 2009). **(C)** Total cytosolic FAD for strains of *A. brasilense* overexpressing Aer. “^∗∗^” indicates statistically significant values (*p*-value < 0.005) relative to the wild type strain. WT(empty vector) corresponds to the wild type strain carrying pRK415, WT(Aer) is the wild type strain carrying pRKAer, Daer(empty vector) is the Δ*aer* mutant derivative carrying pRK415 and Δaer(Aer) is the Δ*aer* mutant derivative carrying pRKAer.

To identify *A. brasilense* chemotaxis receptors with PilZ domains, we conducted a PSI-BLAST using the PilZ domain of Tlp1 (AAT76671.1) as a query. After seven iterations, results converged to seven unique PilZ-containing putative chemotaxis receptors. The most common PilZ containing chemotaxis receptor across all strains of *A. brasilense* identified by the PSI-BLAST is AZOBR_p280026. This chemotaxis receptor is predicted to be membrane anchored and to possess a cytoplasmic N-terminal sensory domain comprised of a PAS/PAC domain followed by, two transmembrane domains, a HAMP domain, a MA, and a C-terminal PilZ domain (Figure 1A). The PilZ domain contains the two strictly conserved arginine residues (R506, R507) required for binding c-di-GMP (Figure 1A) in homologs (Amikam and Galperin, 2006; Ryjenkov et al., 2006; Schirmer and Jenal, 2009) indicating it is likely a functional c-di-GMP effector.

Except for the PilZ domain, the topology and domain organization of AZOBR_p280026 is similar to that of the *E. coli* Aer receptor, which functions in aerotaxis as an energy taxis receptor (Bibikov et al., 2000; Watts et al., 2005). The *E. coli* Aer chemotaxis receptor binds FAD non-covalently within its N-terminal PAS domain and this cofactor binding is required for the receptor energy taxis function. Indeed, the FAD cofactor confers the *E. coli* Aer receptor with the ability to sense changes in redox that occur when cells navigate gradients of oxygen and other cues (Repik et al., 2000). A similar role for FAD non-covalent binding to the *A. brasilense* AerC chemotaxis receptor was previously found (Xie et al., 2010). In both the *E. coli* Aer and the *A. brasilense* AerC, FAD binding depends on a set of conserved residues (Bibikov et al., 1997; Bibikov et al., 2000; Watts et al., 2005; Xie et al., 2010). Sequence analysis indicates that the protein encoded by AZOBR_p280026 contains all of the conserved residues necessary for FAD binding (Figure 1B). In *E. coli* and *A. brasilense*, overexpression of Aer or AerC causes an increase in total intracellular FAD content (Bibikov et al., 1997; Bibikov et al., 2000; Xie et al., 2010). Similarly, when we overexpressed AZOBR_p280026 in *A. brasilense*, the total FAD content of cells increased, suggesting this chemotaxis receptor likely behaves like *E. coli* Aer and *A. brasilens*e AerC (Figure 1C). Given these properties and the function of this receptor in *A. brasilense* aerotaxis (see below), we named it Aer.

### *A. brasilense* Aer PilZ Domain Is Essential for Aerotaxis

**FIGURE 2:**
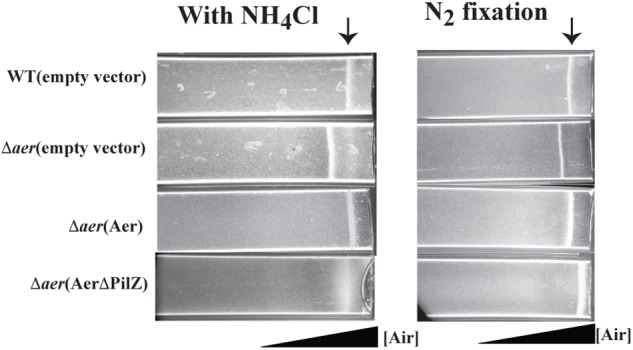
Effect of loss of Aer on oxygen sensing in a spatial assay for aerotaxis. Cells grown in presence of ammonium chloride or grown in the absence of combined nitrogen and under condition of nitrogen (N_2_) fixation before being were placed in a capillary tube. Over 95% of cells are motile under these conditions. An oxygen gradient is generated by diffusion of air into the suspension of motile cells. WT cells accumulate in a tight band at an oxygen concentration optimum for metabolism (arrow). Representative images are the result of 3 biological replicates.

To probe the function of Aer in *A. brasilense* sensing, we created a markerless deletion mutant, named Δ*aer* (Table 1). Given the predicted FAD binding by the *A. brasilens*e Aer receptor and the role of aerotaxis as the strongest behavioral response in this species, we predicted Aer would function as an aerotaxis receptor. To characterize the role of Aer in aerotaxis, we used the spatial gradient assay for aerotaxis and compared the behavior of the wild type strain, the Δ*aer* mutant derivative and its complemented strains expressing either full length Aer or AerΔPilZ that were either grown with ammonium chloride as a nitrogen source or grown under conditions of nitrogen fixation (Figure 2). In the spatial aerotaxis assay, an open-ended capillary tube is filled with motile cells, and an oxygen gradient is established through diffusion of air from the atmosphere into the cell suspension (Wong et al., 1995; O’Neal et al., 2018). Under these conditions, motile *A. brasilens*e cells accumulate in a tight band at a location that corresponds to an oxygen concentration optimum for metabolism (Zhulin et al., 1996; O’Neal et al., 2018). When cells were grown with ammonium prior to being exposed to the gradient, the Δ*aer* mutant cells formed an aerotactic band further away from the air-liquid interface compared to the WT cells (Figure 2, left panel). The aerotaxis defect could be restored by expressing the parental Aer *in trans* from a low copy vector, however, expressing a truncated form of Aer that lacked the C-terminal PilZ domain (AerΔPilZ) failed to restore aerotaxis band formation, though there was cell accumulation at the position of the aerotaxis band should have formed. A similar behavior was observed for cells grown under conditions of nitrogen fixation, with the notable exception that Δ*aer* mutant cells expressing AerΔPilZ accumulated as a tight band closer to the air-liquid meniscus (Figure 2, right panel). We also observed that Δ*aer* cells expressing AerΔPilZ began to clump together and lose motility after band establishment when they were close to the air-liquid interface (at about 3 min), suggesting that cells accumulate at oxygen concentrations that are too high and trigger stress responses (Bible et al., 2015). Together these data show that Aer is required for aerotaxis regardless of nitrogen availability. Our data indicate that cells can form an aerotactic band in the absence of Aer, suggesting other receptors like Tlp1 and AerC can mediate the behavior. In the absence of Aer, the aerotactic band forms further away from the meniscus, suggesting that Aer’s signaling is required to guide cells into regions of higher oxygen concentrations. Strains expressing AerΔPilZ form the aerotactic band at high oxygen concentrations under nitrogen fixation conditions, but the band is abolished when organic nitrogen is available. Together these findings suggest that the contribution of the PilZ domain to Aer signaling during aerotaxis depends on growth (organic nitrogen availability) conditions.

**Table 1:** List of strains used in this study.

Strain	Characteristics	Reference
***E. coli***		
TOP10	General cloning strain	
TOP10 (pk18mobsacB)	TOP10 cells carrying pk18mobsacB, KanR, SucS	
TOP10 (pk18mobsac_ AerUpDown)	TOP10 cells carrying pk18mobsacB with Upstream and Downstream AZOBRv2_p28002 sequences, KanR, SucS	This Work
TOP10 (pRK415)	TOP10 cells carrying pRK415, TetR	This Work
TOP10 (pRKAer)	TOP10 cells carrying pRK415 encoding AZOBRv2_p280026 (*aer*), TetR	This Work
TOP10 (pRK AerΔPilZ)	TOP10 cells carrying pRK415 encoding truncated AZOBRv2_p280026 (*aer*), lacking the C-terminal PilZ domain, TetR	This Work
***A. brasilense***		
Sp7	Wild Type, AmpR	Tarrand et al., 1978
**WT(empty vector)**	Sp7 carrying the vector pRK415	Russell et al., 2013
*Δaer*	Sp7 with AZOBRv2_p280026 (*aer*) deleted	This work
*Δaer*(Aer)	Δaer with Aer expressed from pRK415, AmpR TetR	This work
Δ*aer(*AerΔPilZ)	ΔAer with truncated Aer expressed from pRK415, AmpR TetR	This work


As expected for its role in aerotaxis, cells lacking Aer or expressing the variant lacking PilZ did not have any defect in chemotaxis toward organic acids (Supplementary Figure S1), indicating that Aer is dispensable in chemotaxis.

### Aer Contributes to Competitive Root Surface Colonization

**FIGURE 3:**
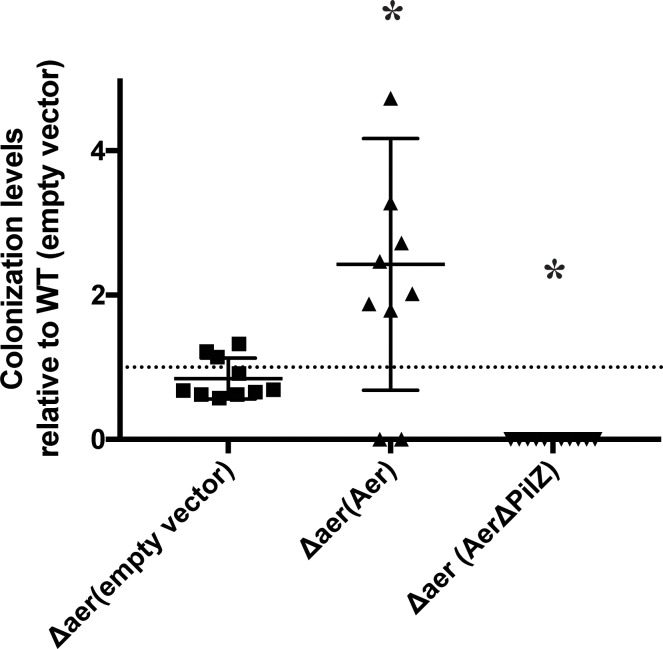
Colonization efficiency of wheat seedling roots by *A. brasilense* wild type and mutant strains derivative lacking Aer and expressing an empty vector control, Aer or AerΔPilZ. The colonization index was the log_10_(strain output/strain input), where the number of CFU extracted from roots after incubation was normalized to the number of CFU measured in the inoculated input. The dotted line value at 1 indicates the colonization index of the WT(empty vector). Points above the line are representative of a higher colonization efficiency, while points below the line are indicative of a lower colonization efficiency. ^∗^ indicates significant difference from WT(empty vector) (*p*-value < 0.05).

Given the role of Aer in aerotaxis, we next tested how this response contributed to colonization of wheat (*Triticum aestivum*) root seedlings. The Δ*aer* strain colonized the roots at levels similar to wild type, when inoculated alone while the Δ*aer* strain expressing the parental Aer colonized at higher levels than the wild type strain (Figure 3). These differences in the colonization ability of the strains inoculated alone were unexpected and could not be attributed to changes in the level of motility between the strains. We hypothesized that functional Aer would provide a competitive advantage in colonization of the root surface and tested this idea by inoculating the wild type and the Δ*aer* strains in competition at a 1:1 ratio (See “Materials and Methods”). After 5 days, the strains were recovered from homogenized wheat roots at a ratio of wild type to Δ*aer* mutant of 92:8. These findings indicate that the Δ*aer* mutant is severely impaired in its ability to compete with the wild type strain for wheat root surface colonization despite being able to colonize the root surfaces when inoculated alone. Surprisingly, the Δ*aer* strain expressing AerΔPilZ was severely impaired in colonization of the root surfaces when inoculated alone (Figure 3). These observations hint at a complex contribution of Aer and PilZ-mediated signaling in the vicinity of the roots.

### Aer Signaling Is Integrated With That of Other Chemotaxis Receptors in a PilZ-Dependent Manner

**FIGURE 4:**
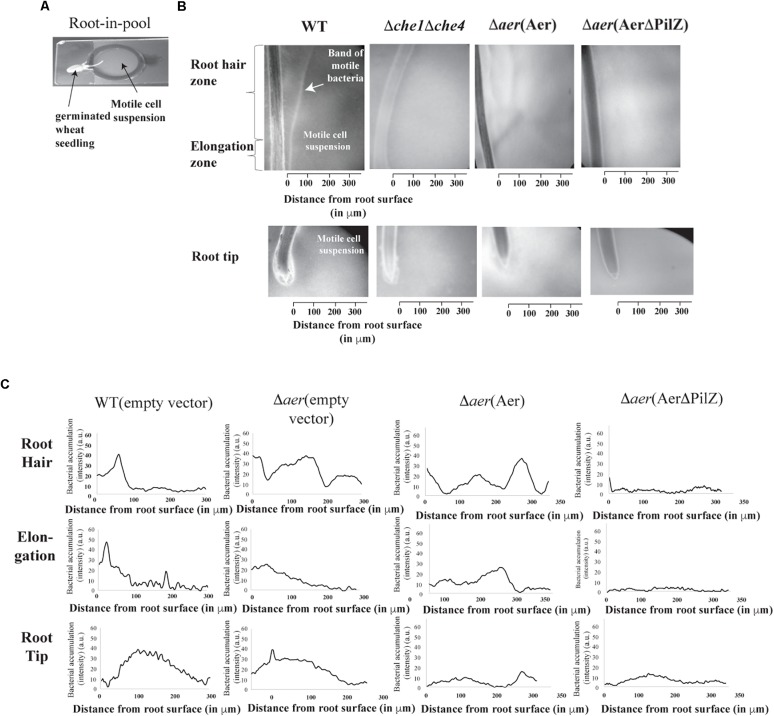
*A. brasilense* accumulation around wheat roots using a root in pool assay. The root in pool consists of a divet slide with an attached O-ring. The pool of bacteria is placed in the O-ring and the root is dropped in. The cell-root suspension is covered with a coverslip and 4× magnification on a phase contrast scope is used to film **(A)**. This set up allows for monitoring of bacteria-root interactions at the root hair, elongation and tip zones **(B)**. Bacterial cell accumulation (as image intensity in arbitrary units) away from root zones indicated in the different strains of *A. brasilense* **(C)**. Shown are representative image intensity plots (bacterial accumulation) of five biological replicates of the strains.

To gain further insight into the apparent complex contribution of *A. brasilense* Aer and Aer PilZ in wheat root surface colonization, we sought to develop an assay permitting real time observation of chemotaxis responses, which we call the root-in-pool assay. In this assay, a 5-day old germinated wheat seedling’s root is placed into a pool of concentrated motile cells (Figure 4A). The immediate response of motile bacteria to the presence of the roots is recorded for up to 5 min and the preferential accumulation of bacteria in different regions around the roots can be inferred from image intensity analysis (see “Materials and Methods”). When exposed to wheat roots, the wild type *A. brasilense* cells accumulated in a tight band away from the root hair (129 ± 18 μm) and elongation (80 ± 7 μm) zones (Figure 4B), but they depleted a region of about 100 μm out from the root tip (Figure 4C). This response was stable for at least 10 min and was strikingly similar to aerotaxis band formation. To further establish that the response seen was a chemotaxis-dependent response, we performed the same assay with a motile, but non-chemotactic strain of *A. brasilense* (Mukherjee et al., 2016). This strain was motile but failed to accumulate in any region around the roots indicating that the accumulation of motile cells as a band is a chemotactic response (Figure 4B). Cells lacking Aer did not accumulate in a tight band in either the root hair or elongation zones. Instead, local depletions of cells away from the root hair zone were followed by a broad accumulation at some distance from the root hairs, in a pattern that was distinct from that of the wild type cells (Figure 4C). In the elongation zone, a broad but weak accumulation of cells close to the root surface could be detected. These results suggest that Aer is required for the discrete accumulation of cells in bands in the vicinity of the root hair and root elongation zones. The Δ*aer* strain was not repelled from the root tip and accumulated closer to the root tip (Figure 4C). The non-chemotactic strain showed no response in this region. Expressing full length Aer from a plasmid did not restore the accumulation and banding pattern seen for wild type cells in the root hair and elongation zones, suggesting that the chemotaxis response observed in the wild type integrates signaling from other chemotaxis receptors in addition to Aer. While this is not unexpected given the chemical complexity of root exudates (Walker et al., 2003), the behavioral differences likely account for the surprising difference in the ability of the strains to colonize the root surface when inoculated alone as seen above. Complementing with full length Aer abolished the small accumulation of the cells at the root tips and thus partially rescued the phenotype, suggesting a greater contribution of Aer to this particular response. The Δ*aer*(AerΔPilZ) strain was not able to accumulate as tight bands of motile cells at any location and showed no response in the vicinity of the root tips, despite being fully motile. Cells from this strain remained distributed around the roots without any specific accumulation pattern which was similar to that of a non-chemotactic mutant (Figure 4B). Lack of PilZ in Aer thus abolishes the ability of cells to sense chemical gradients originating from the roots, suggesting a critical role of PilZ from Aer in ensuring chemotaxis signaling function.

## Discussion

The plant rhizosphere is a nutrient rich environment compared to the surrounding soil and it is characterized by a plethora of chemical gradients (Bais et al., 2006). Bacterial chemotaxis is functionally enriched in the genomes of soil and rhizosphere bacteria suggesting it provides a critical competitive advantage (Buchan et al., 2010). With its 51 chemotaxis receptors and 4 chemotaxis signaling systems, *A. brasilense* is a model representative of the complex signaling diversity found in bacteria that colonize the rhizosphere of various plants. Of the 51 chemotaxis receptors encoded within the *A. brasilense* genome, Tlp1 and AerC have been previously characterized (Greer-Phillips et al., 2004; Xie et al., 2010). Interestingly, both modulate aerotaxis responses but only Tlp1, and not AerC, is critical for wheat root surface colonization. Here we show that *A. brasilense* Aer, a membrane anchored chemotaxis receptor that is likely to bind FAD, modulates aerotaxis, as expected from its similarity with *E. coli* Aer (Bibikov et al., 2000; Watts et al., 2005). Evidence obtained here also suggests that *A. brasilense* Aer is critical to adjust the position of the cells in a gradient in response to elevated oxygen concentrations.

Given that *A. brasilense* Aer likely binds FAD as a cofactor, it likely functions as a redox sensor rather than an exclusive aerotaxis receptor, i.e., it monitors changes in redox that alter the flux of FAD/FADH2 in the cell (Repik et al., 2000; Watts et al., 2004). Further evidence for this broader role includes the complex, albeit unclear, role of Aer in sensing chemical gradients emanating from wheat roots in the root-in-pool assay. Chemicals exuded by roots are likely to affect metabolism and thus the intracellular redox status of the cells, which would be sensed by Aer and other energy taxis receptors.

Here we also show that PilZ domain contributes to Aer function and likely, to the integration of Aer signaling with other chemotaxis receptor signaling input. Chemotaxis signaling receptors are organized as large arrays of interacting trimers of chemotaxis receptor dimers (Briegel et al., 2014; Frank et al., 2016). This organization is required for signal integration, amplification and for chemotaxis signal sensitivity. This array organization is universally conserved in bacteria (Buchan et al., 2010) and accounts for the summative operation of chemotaxis receptor information to produce an integrated response (Sourjik and Berg, 2002, 2004). We hypothesize that it is this computation of signals from diverse receptors that explain some of the puzzling effects we have observed when deleting PilZ from Aer. We show that lack of Aer yields aerotaxis defects that are fully complemented by expression of the parental Aer *in trans*, suggesting the defect is due solely to lack of signaling by Aer. In the absence of Aer, other chemotaxis receptors, for example Tlp1 and AerC, integrate signaling information together with that of other chemotaxis receptors to produce a response that ultimately guide the cells in the oxygen gradient to form a band at a specific position. These aerotaxis defects are not rescued by expressing an Aer variant lacking the PilZ domain and in fact produce additional defects that depend on the environmental conditions: in presence of ammonium, but not under conditions of nitrogen fixation, PilZ-mediated Aer function was critical since no band formed. These results suggest that (i) a different set of chemotaxis receptors contribute to aerotaxis signaling and response depending on the nitrogen nutrition status and, (ii) that PilZ from Aer is required for the ability of this receptor to integrate signaling with other chemotaxis receptors. *A. brasilense* AerC is a major aerotaxis receptor under conditions of nitrogen fixation but not under conditions when ammonium chloride as nitrogen source (Xie et al., 2010), an observation fully consistent with the defects seen here. The effect of Aer PilZ on integration of Aer signaling with other, unknown receptors, is most strikingly observed in response to the complex gradients exuded by wheat roots. Under these conditions, functional PilZ was essential for the ability of cells to detect chemical gradients and to display any chemotaxis response. This, in turn suggests that c-di-GMP metabolism and its effect on receptors such as Aer is likely a critical factor for chemotaxis and competitiveness in the rhizosphere in this bacterial species. *A. brasilense* encodes 34 diguanylate cyclases and phosphodiesterases that are predicted to sense a variety of cues to modulate c-di-GMP metabolism (Mata et al., 2018). Oxygen is a major regulator of c-di-GMP metabolism in *A. brasilens*e (Russell et al., 2013), and findings here indicate that c-di-GMP signaling and integration with chemotaxis is likely critical for the ability of *A. brasilense* to detect chemical gradients in the rhizosphere. This hypothesis is further supported by the observation that Tlp1, which is also a PilZ-domain containing chemotaxis receptor plays a key role in root surface colonization (Greer-Phillips et al., 2004).

## Materials and Methods

### Bacterial Strains Growth and Maintenance

Wild type *A. brasilense* (Sp7) and its derivative Δ*aer* were maintained on Minimal Medium for *A. brasilense* (MMAB) supplemented with nitrogen (20 mM) and malate (10 mM) (16). All strains were maintained on 1.5% agar with appropriate antibiotics [ampicillin (200 μg/ml), tetracycline (10 μg/ ml), kanamycin (30 μg/ml)] (Table 1).

### Identification of Genes Encoding Putative Chemotaxis Receptors With Additional Domains and PAS Sequence Alignment

The PilZ domain of Tlp1 (AAT76671.1) was used in a PSI-BLAST against all *A. brasilense* species to identify any receptors containing C-terminal PilZ domains. Each putative protein was verified using SMART domain search. Genes encoding each protein were identified in the Sp7 genome (GenBank project PRJNA265779 ). Residues 25-139 of the PAS domains of K. pneumoniae NifL (AAB26403.1), residues 8-129 of the PAS domain of the *E. coli* Aer (AYG17963.1), and residues 85-329 of the *Methylococcus capsulat*us MmoS (AAP80772.1) were aligned with residues 9-121 of the PAS domain of *A. brasilense* Aer using the COBALT multiple sequence alignment in NCBI (Papadopoulos and Agarwala, 2007).

### Generation of Markerless Deletion of Aer

The upstream and downstream sequences of AZOBR_p280026 were amplified using the primers listed in Table 2 and then cloned into the pk18mobsacB vector using restriction digestion. The plasmid containing the upstream and downstream sequences was confirmed via sequencing and transformed into *E. coli* TOP10. The plasmid was then transferred into WT *A. brasilense* via triparental conjugation, as previously described (Gullett et al., 2017) and selected for on MMAB with 10 mM malate and kanamycin (30 μg/ml).

**Table 2:** Primers used in this study.

Primer name	Sequence
AZOBRv2_p280026-EcoR1-F	5′-CCCGAATTCACAATAGGGGCGAAGCGC-3′
AZOBRv2_p280026-ovl-R	5′-GCCGCCGTTGCGGATCTCGGCGTAGATGC GCTCGGC-3′
AZOBRv2_p280026-ovl-F	5′ GAGATCCGCAACGGCGGCGCCATGCT GAGTGGGATC-3′
AZOBRv2_p280026-Sal-R	5′-CCC GTCGAC CTTCGCCGCGACCGAGCC-3′
Aer complementation Fwd HindIII	5′-AAGCTTATGCGCGATAATGGACCGGT-3′
Aer complementation Rev BamHI	5′-GGATCCTCAGGCCGCCGCCGTCGCCA-3′
AerΔPilZ complementation Fwd HindIII	5′-AAGCTTATGCGCGATAATGGACCGGT-3′
AerΔPilZ complementation Rev BamHI	5′-GGATCCTCAGGCGTCGCTGGTGGAGGTGC-3′


Single recombinants were screened on MMAB plates containing kanamycin (30 μg/ml). Once single recombinants were identified, they were passaged through TY without antibiotic selection 3–5 times. With every passage, serial dilutions were performed and plated on TY (Tryptone-Yeast) plates containing 10% sucrose (w/v). Colonies that appeared on TY containing 10% sucrose were rescreened on TY and TY with kanamycin (30 μg/ml) (Gullett et al., 2017). Colonies with suspected gene deletions were PCR confirmed.

### Functional Complementation of the Δaer Mutation

The gene encoding full length Aer or truncated Aer (AerΔPilZ) was amplified and cloned into pRK415 using restriction digestion and sequencing confirmed and transformed into *E. coli* TOP10 cells for maintenance. The plasmids were transferred into Δ*aer* using triparental conjugation as described above.

### FAD Quantification

The 50 ml of wild type cells or Δaer expressing the parental Aer were grown to OD600 = 0.8 and pelleted. Intracellular FAD was extracted as previously described in Watts et al. (2004) and Xie et al. (2010) using 0.6 N perchloric acid and sodium bicarbonate. The cell extract was combined with 23 mM D-alanine, 7.5 μg horseradish peroxidase, and 25 μM luminol and the reaction was initiated with apo-D-amino acid oxidase 1 mg/ ml and luminescence was measured every second for 1 h. Peak luminescence readings (18 min) were used for FAD quantification.

### Chemotaxis Soft Agar Plate Assays

*Azospirillum brasilense* strains were grown in MMAB supplemented with nitrogen (20mM) and malate (10mM) to OD600 = 0.8. One ml of the culture was centrifuged and washed with Che buffer (1.7 g L^-1^ dipotassium phosphate, 1.36 g L^-1^ monopotassium phosphate; 0.1 mM EDTA), and 10 microliters was inoculated into MMAB containing 0.3% agar. Chemotaxis rings were measured 72 h post-inoculation.

### Spatial Gradient Assay for Aerotaxis

*Azospirillum brasilense* strains were grown and washed as previously described. Washed cells were used to fill an optically flat capillary tube. The capillary tube was equilibrated in N_2_ for 3 min. N_2_ was then shut off and air was introduced, and band formation was monitored using a 4X objective of a phase contrast Nikon E200 microscope (O’Neal et al., 2018). Images were acquired using a C-mounted Nikon Coolpix digital camera. Distance of band formation of the meniscus and band width was quantified using the measure tool in FIJI. Distance from the meniscus to the center of the band at mid-height of the capillary tube. All measurements were performed at 180 s after air introduction.

### Root-in-Pool Assay

Wheat seeds were sterilized with successive washes of bleach, 70% ethanol containing 1% Triton X-100, and sterile water. After sterilization, seeds were placed in a sterile petri dish containing a small volume of sterile water and placed in the dark. After 48 h, plates were placed in the light and allowed to grow for another day. All assays were performed on 5-day old germinated seeds. *A. brasilense* strains were grown in MMAB with nitrogen (20 mM) and malate (10 mM). OD600 = 0.4 and washed with chemotaxis buffer as previously described. Cells were starved in chemotaxis buffer (1.7 g L^-1^ dipotassium phosphate, 1.36 g L^-1^ monopotassium phosphate; 0.1 mM EDTA) for 30 min before exposure to the root. A black washer with a slit cut in it was secured to a depression slide using craft glue. The germinated root was placed in the slit and 400 μL of washed bacteria was used to fill the chamber created by the dip and washer, and then a cover slip was used to cover the pool chamber. All root-in-pool assays were recorded using the 4x objective of a Nikon E200 phase contrast microscope equipped with Nikon Coolpix digital camera. Assays were filmed for 3 min. FIJI was used for all image and video analysis. All root-in-pool assay videos were converted to 8-bit and background subtraction was performed. Intensity plots were generated out from the root to determine bacterial accumulation. Bacterial accumulation distance from the root surface was measured at the mid-point of each root zone. The average accumulation distance of five biological replicates is reported.

### Wheat Root Colonization Assay

Wheat seeds were germinated as previously described. *A. brasilense* strains were grown to OD600 = 0.4, washed and suspended in 400 μl of ChE buffer (1.7 g L^-1^ dipotassium phosphate, 1.36 g L^-1^ monopotassium phosphate; 0.1 mM EDTA). Serial dilutions were performed to determine the number of bacteria inoculated. Plant growth chambers were filled with 50 ml of semi-solid Fahraeus media (Gosta, 1957) (0.9 mM CaCl_2_^.^H_2_O, 0.5 mM MgSO_4_, 0.7 mM KH_2_PO_4_, 0.8 mM Na_2_HPO_4_^.^7H_2_O, 20 μM Fe-citrate, 33 μg/l, a pinch of Na_2_MoO_4_⋅H_2_O, pH 7.0, 4 g agar per liter), and four germinated seedlings were planted in the chamber. Twenty microliters of the bacterial suspension were inoculated into the center of the growth chamber, and after 5 days roots were harvested, homogenized, and serial dilutions were performed. Student’s unpaired *t*-test was performed to determine if colonization levels were significantly different (*p*-value = 0.05). Root colonization was calculated as a colonization index. The colonization index for an individual strain was the log_10_(strain output/strain input), where the number of CFU extracted from roots after incubation was normalized to the number of CFU measured in the inoculated input. Competition assays were performed by inoculating equal amounts of WT and Δ*aer* cells (confirmed by CFU and OD600 readings). After 5 days CFU counts were performed and 30 colonies from each replicate were screened for deletion of AZOBRv2_p280026 using primers in Table 2.

### Statistical Analysis

Average values from at least three independent experiments performed in duplicate and performed one-way analysis of variance (alpha level, 0.05), followed by pairwise two-sample *t*-tests assuming equal variances (alpha level, 0.05) using Prism (version 6) software (GraphPad Software Inc., San Diego, CA, United States).

## Data Availability

All datasets generated for this study are included in the manuscript and/or the Supplementary Files.

## Author Contributions

SA and LO’N performed the experiments. LO’N and GA designed the experiments, analyzed data and wrote the manuscript.

## Conflict of Interest Statement

The authors declare that the research was conducted in the absence of any commercial or financial relationships that could be construed as a potential conflict of interest.
