# Supplementary material for: A PilZ-Containing Chemotaxis Receptor Mediates Oxygen and Wheat Root Sensing in Azospirillum brasilense
Source: Front Microbiol. 2019 Mar 1;10:312. doi: 10.3389/fmicb.2019.00312 (PMC6406031; doi:10.3389/fmicb.2019.00312)
Supplement: Supplementary file 1 [file Data_Sheet_1.pdf]

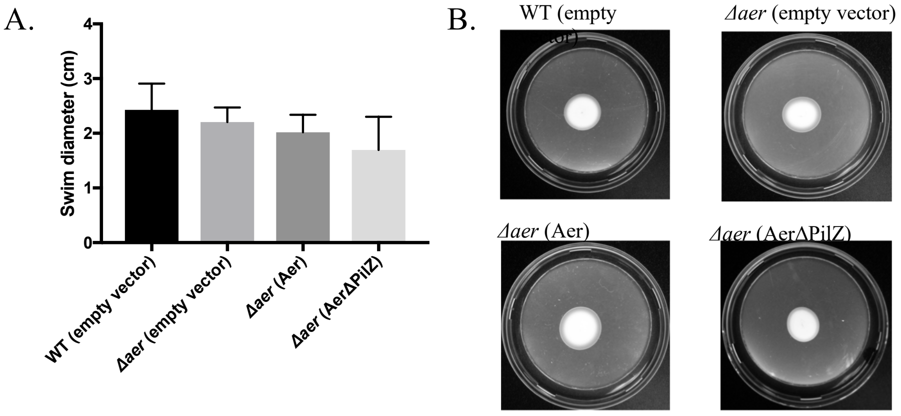

Fig S1: Chemotaxis in a soft agar plate containing 10mM malate. A) Average chemotaxis ring diameter of cells in a soft agar plate containing malate with standard deviation. Given in the average of 3 biological replicates. B) Representative images of WT(empty vector), Δ*aer*(empty vector), and Δ*aer*(pRK Aer), and Δ*aer*(pRKAerΔPilZ).
